# Effectiveness of a specific manual approach to the suboccipital region in patients with chronic mechanical neck pain and rotation deficit in the upper cervical spine: study protocol for a randomized controlled trial

**DOI:** 10.1186/s12891-017-1744-5

**Published:** 2017-09-05

**Authors:** Vanessa González Rueda, Carlos López de Celis, Martín Eusebio Barra López, Andoni Carrasco Uribarren, Sara Castillo Tomás, Cesar Hidalgo García

**Affiliations:** 1 Rehabilitation Service Baix Llobregat Centre, DAP Costa de Ponent, Catalan Institute of Health, Barcelona, Spain; 2 Jordi Gol Institute of Research on Primary Health Care, Barcelona, Spain; 3 Faculty of Medicine and Health Sciences, International University of Catalonia, Barcelona, Spain; 4 Faculty of Health Sciences, University of Zaragoza, Zaragoza, Spain; 5 FREMAP, Mutual Society for Work-related Injuries and Occupational Diseases, Arnedo, Spain

**Keywords:** Range of motion, Cervical vertebrae, Atlantoaxial joint, Suboccipital muscle inhibition

## Abstract

**Background:**

Mechanical neck pain is a highly prevalent problem in primary healthcare settings. Many of these patients have restricted mobility of the cervical spine. Several manual techniques have been recommended for restoring cervical mobility, but their effectiveness in these patients is unknown. The aim of the present study is to compare the effectiveness of two types of specific techniques of the upper neck region: the pressure maintained suboccipital inhibition technique (PMSIT) and the translatory dorsal glide mobilization (TDGM) C0-C1 technique, as adjuncts to a protocolized physiotherapy treatment of the neck region in subjects with chronic mechanical neck pain and rotation deficit in the upper cervical spine.

**Methods:**

A randomized, prospective, double-blind (patient and evaluator) clinical trial. The participants (*n* = 78) will be randomly distributed into three groups. The Control Group will receive a protocolized treatment for 3 weeks, the Mobilization Group will receive the same protocolized treatment and 6 sessions (2 per week) of the TDGM C0-C1 technique, and the Pressure Group will receive the same protocolized treatment and 6 sessions (2 per week) of the PMSIT technique. The intensity of pain (VAS), neck disability (NDI), the cervical range of motion (CROM), headache intensity (HIT-6) and the rating of clinical change (GROC scale) will be measured. The measurements will be performed at baseline, post-treatment and 3 months after the end of treatment, by the same physiotherapist blinded to the group assigned to the subject.

**Discussion:**

We believe that an approach including manual treatment to upper cervical dysfunction will be more effective in these patients. Furthermore, the PMSIT technique acts mostly on the musculature, while the TDGM technique acts on the joint. We expect to clarify which component is more effective in improving the upper cervical mobility.

**Trial registration:**

ClinicalTrials.gov NCT02832232. Registered on July 13th, 2016.

## Background

Most cases of pain in the neck region are of mechanical origin [1]. The annual incidence of episodes of mechanical neck pain is estimated at 12 per 1.000 subjects with a primary care medical appointment [2], making it one of the main health problems seen by physiotherapy units in primary care [3]. Its annual prevalence has been estimated as ranging between 16.7% and 75.1% [4]. An annual prevalence of 19.5% has been calculated in the Spanish population [5]. It lasts for 6 months or longer in 14% of cases [6], leading to difficulties not only at work but also in everyday life, at home and in leisure activities [7]. The healthcare costs generated are high, mostly as a result of compensation for sick leave [8, 9].

The most common symptom is pain in the neck region, which may be associated with dizziness, light-headedness, restricted movement [10] and manifestations of stress [11]. A large proportion of the movement of the cervical spine takes place in the C1-C2 segment, where up to 50% of the total rotation of the cervical spine occurs [12].

There are several techniques to restore cervical mobility, but few of them comply with the guidelines of the IFOMPT (International Federation of Orthopaedic Manipulative Physical Therapists) on safety and effectiveness in treatment of hypomobility of the upper cervical spine, avoiding positions at the end of the range of cervical movement, especially in rotation and extension [13], and there is limited scientific evidence for their effectiveness. The pressure maintained suboccipital inhibition technique (PMSIT) [14] and the translatory dorsal glide mobilization technique in grade III of the atlanto-occipital joint (TDGM C0-C1) described by Olaf Evjenth [15] comply with these recommendations and have been used in previous studies [16], although there is no evidence for their effects on the range of mobility in patients with chronic mechanical neck pain.

Our objective is to compare the effectiveness of the pressure maintained suboccipital inhibition technique and the translatory dorsal glide mobilization technique in grade III of the atlanto-occipital joint as adjunct treatments for physiotherapy in subjects with chronic mechanical neck pain and rotation deficit in the upper cervical spine.

## Methods

### Study design

**Fig. 1:**
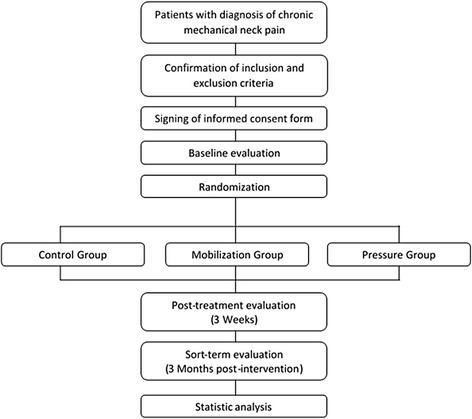
Consort Diagram: Flow of the participants throughout the study

A randomized, prospective, double-blind (patient and evaluator) clinical trial. The therapist cannot be blinded due to the use of manual techniques. Figure 1 shows a diagram with the different phases of the study.

### Sample size calculation

The required calculation of the sample size was performed for all outcome variables, for a two-tail test, using a web application (Granmo v7.12) from the Mar Institute of Medical Research Foundation (https://www.imim.es/ofertadeserveis/software-public/granmo/). The statistics needed for the calculation were determined based on three studies: the pilot study by González-Rueda et al. [16] provided statistical data about pain intensity in a similar population and with similar techniques to those used in this study, and those by Izquierdo et al. [17] and Dunning et al. [18] comparing different manual techniques in patients with chronic mechanical neck pain provided statistical data about Neck Disability Index (NDI), range of active mobility an Headache Impact Test. The highest value obtained, using an alpha risk of 0.05 and a beta risk of 0.20 with an estimated follow-up loss of 15%, was 26 subjects per group. As there will be three study groups (the Control Group, the Mobilization Group and the Pressure Group) the total sample size will be 78 subjects.

### Study population. Sample recruitment

The subjects for this study will be recruited in the Sant Ildefons Rehabilitation Service in Cornellà de Llobregat (Barcelona), a public Primary Care centre of the Catalan Institut of Health. All patients referred to this service must be seen by a specialist in Physical Medicine and Rehabilitation before receiving treatment. All those diagnosed with chronic mechanical neck pain (of more than 3 months duration) and classified in Grade I or Grade II according to the classification of the Neck Pain Task Force [19] during that appointment will be referred to a physiotherapist, who will check whether they meet the other inclusion and exclusion criteria before offering them the opportunity to participate in the study.

The inclusion criteria will be: subjects diagnosed with chronic mechanical neck pain, who are over 18 years old and with a positive result in the flexion-rotation test corresponding to a rotation deficit. The flexion-rotation test (FRT) will be considered as positive if in a position of maximum cervical flexion, there is a difference of 10 degrees or more in the rotation in one direction compared to the contralateral direction, or if there is a cervical rotation of less than 32 degrees measured with the CROM [12, 20].

The exclusion criteria will be: contraindication to manual therapy, post-traumatic neck pain or red flags according to Rushton et al. [13], an inability to maintain the supine position, use of pacemakers, an inability to perform the FRT, language difficulties that hinder understanding of informed consent or completion of the questionnaires necessary for this study, and subjects with litigation or lawsuits pending.

Patients who meet all inclusion criteria and none of the exclusion criteria will be offered the opportunity to participate in the study and provided with all the relevant information verbally and in writing. They will be told that they will be randomly distributed among the study groups and that each group will be treated with different techniques, all of which are appropriate for their condition, and that the objective of the study is to determine which technique produces the best results. The patients will not be informed about which group they have been assigned to in order to maintain blinding in this regard.

If they decide to participate they will be asked to sign an informed consent form and the same physiotherapist responsible for recruitment will perform the baseline assessment.

### Randomization

A list of consecutive numbers (from 1 to 78) will be generated prior to recruitment, and each of these numbers will be assigned randomly to one of the three study groups (Control Group, Mobilization Group and Pressure Group) using a computer program (www.random.org).

Without any prior knowledge of the list generated, the physiotherapist responsible for recruitment and initial assessment will assign a consecutive number to each subject participating in the study.

The physiotherapist performing the intervention will be the only one with access to the list generated by the computer program. This physiotherapist cannot be blinded due to using manual techniques and he/she will know which group the subject has been assigned to by referring to the number assigned by the evaluator.

The concealment and blinding of the assignment will therefore be assured, since the physiotherapist performing the recruitment will not know which group the subject will be assigned to.

### Variables studied

The physiotherapist responsible for recruitment (Master’s Degree in Orthopaedic Manual Therapy and 7 years of experience in this field) will perform all the evaluations for this study, and will remain blinded as regards the group that the subject has been assigned to at all times.

Table 1 shows the sociodemographic variables and the values that each one may have. The following variables: age, sex, duration of symptoms, work, work with lifting, work with sustained focal distance, housework, use of mouthguard, presence of dental prosthesis, visual problems, presence and frequency of headaches and analgesic and anti-inflammatory medication will be recorded at the beginning of treatment by anamnesis.

**Table 1:** Independent sociodemographic variables

Variable	Values
Age	Years
Sex	Man/Woman
Duration of symptoms	Months
Work	Employed/Unemployed
Work with lifting	Yes/No/Varied
Work with sustained focal distance	Hours
Housework	Hours
Mouthguard	Yes/No
Dental prosthesis	Yes/No
Visual problems	Yes/No
Headaches	Yes/No
Headache frequency	Daily - Weekly - Monthly - Quarterly - Annual
Medication	Yes/No

Table 2 shows the outcome variables, the evaluation period for each one and the measuring instrument used. All evaluations will be performed by a single physiotherapist, who will also be responsible for the recruitment, and who will be blinded throughout the evaluation period as regards the group assigned to each subject.

**Table 2:** Outcome variables

Outcome variables	Evaluation period			Measuring instrument
	Baseline	Post-treatment	Three months post-treatment	
Intensity of neck pain (main outome)	X	X	X	VAS
Neck disability	X	X	X	NDI
Overall cervical range of motion	X	X	X	CROM
Upper cervical range of motion	X	X	X	CROM
Headache intensity	X	X	X	HIT-6
Rating of clinical change		X	X	GROC scale

Main outcome will be the intensity of pain in the cervical region measured with a visual analog scale (VAS) from 0 to 100 mm in length, with the extremes defined as “no pain” (0) and “the worst pain imaginable” (100) and without any intermediate points. The test-retest reliability has proven to be good (ICC 0.92) [21].

Neck disability will be measured using the Neck Disability Index (NDI). This is a self-applied questionnaire consisting of 10 sections with 6 possible answers representing six progressive levels of functional disability, rated from 0 to 5, with 0 being the first level and 5 the last level of each section. The total score ranges from 0 to 50 points, with higher scores indicating greater disability. At least 8 of the 10 sections must be answered for the score to be calculated. The test-retest reliability has proven to be adequate (ICC 0.97) and it has been validated for the Spanish language [22].

The overall range of active mobility in the cervical region will be evaluated in a sitting position with the vertical back resting on the backrest of the chair [23]. Flexion, extension, right and left inclination and left and right rotation will be measured. A CROM device with good intra- and interexaminer reliability (ICC > 0.80) [24] will be used in all the measurements. Two measurements will be made for each movement, and the result will be the mean of the two measurements.

The range of active mobility in flexion and extension of the upper cervical region will be measured in a standing position with the back against a wall. The flexion-rotation test [25, 26], which has proven to have high levels of intra- and interexaminer reliability (ICC 0.98) [27] will be used to measure the range of motion in rotation in the upper cervical region. To perform the FRT, the subjects position themselves in the supine position and the evaluator passively takes the patient’s cervical spine to its maximum flexion and then rotates the head to the right and left side with the occiput resting against the evaluator abdomen. The movement stops at whichever situation occurs first, either the subject presents symptoms, or the evaluator reaches the end of the range of motion and finds a hard end feel [12]. As in the measurement of overall cervical mobility, a CROM device will be used for all the movements of the upper cervical region, and two measurements will be performed for each movement, with the result being the mean of the two measurements.

Headache intensity is measured by the validated Spanish version of the HIT-6 (Headache Impact Test) questionnaire [28]. This questionnaire consists of 6 items (intensity of pain, social functioning, role functioning, vitality, cognitive functioning and psychological disorder), which each have five response options (Never: 6 points, Rarely: 8 points, Sometimes: 10 points, Very often: 11 points, Always: 13 points), with a score range of 36-78 points. It has proven to have good test-retest reliability (ICC 0.80) [29, 30].

The patient’s rating of clinical change will be evaluated with a Global Rating of Change Scale (GROC scale) [31]. This is a scale of 15 items, of which 7 are improvement and 7 are deterioration, and with 1 central item with no clinical change. Values from the fourth item of improvement or deterioration will be considered clinically significant, values between the three item of improvement and the three item of deterioration will be considered as no clinically significant changes [32]. The test-retest reliability has proven to be good (ICC 0.90) [33].

Additionally, changes in medication (more, less or the same dose) and the presence of exacerbations and/or recurrence will be recorded by anamnesis 3 months after the treatment period. Exacerbation is considered the isolated process of increased pain which patients control themselves using the patterns learned, and exacerbations requiring further medical advice will be considered recurrences.

### Intervention

Intervention will be provided by a different physiotherapist (PhD, with more than 13 years of experience in the field of Orthopaedic Manual Therapy) who was the only person having the random allocation list and implemented the treatment according to the group assigned to the participant’s number by the computer program, blinded to the results of the assessment.

The three groups will receive a common protocolized treatment and additionally, the translatory dorsal glide mobilization technique (TDGM) will be applied to the Mobilization Group and the pressure maintained suboccipital inhibition technique (PMSIT) will be applied to the Pressure Group.

#### Control group procedure

The Control Group will only receive the protocolized intervention in 15 daily sessions (Monday through Friday) for 3 weeks. Each session consists of the application of surface thermotherapy (20′ of infrared), an educational discussion (10′ of education about the disease and movement management in the initial sessions, and to answer new questions raised by the patients in subsequently sessions) and kinesitherapy under the physiotherapist’s supervision (30′ of self-stretching of cervical muscles and dorsal flexibility exercises). At the end of the 3 weeks of treatment, patients are instructed to perform self-stretching and dorsal flexibility exercises at home until the final evaluation at 3 months.

#### Mobilization group procedure

The Mobilization Group will receive the same protocolized treatment and the same instructions as the Control Group, and will also receive 6 sessions (two sessions per week on alternate days) of the translatory dorsal glide mobilization technique (TDGM C0-C1). The technique will be applied for 5 min per session in consecutive cycles of dorsal pushes for about 15 s resting for about 3-5 s, until the first marked resistance felt by the therapist has been exceeded, which would be equivalent to grade III according to the Kaltenborn’s diagram of degrees of movement [34]. Usually this technique is not painful, nevertheless when patient refers pain, pressure is adjusted until a non painful sensation.

**Fig. 2:**
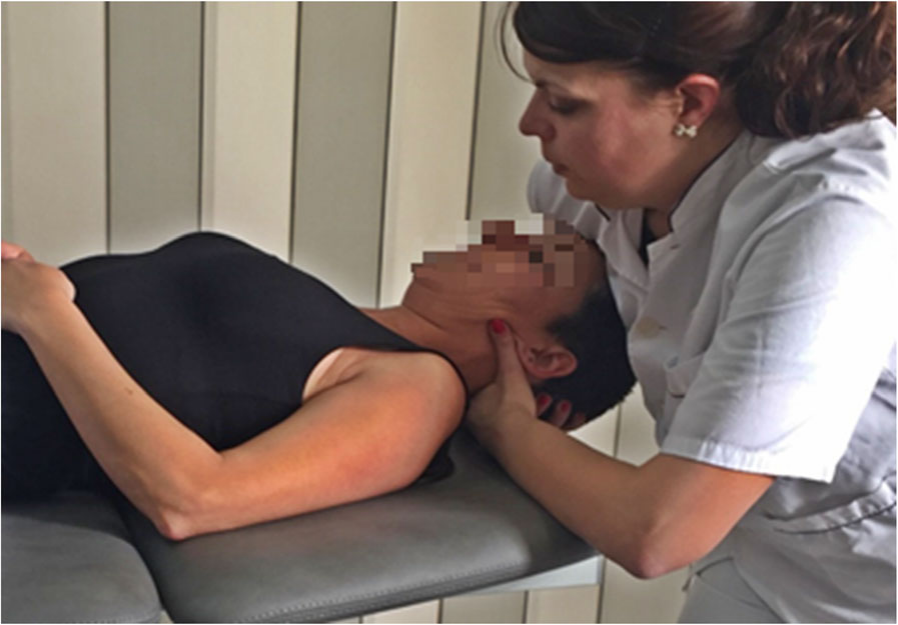
Suboccipital translatory dorsal glide mobilization (TDGM) C0-C1 technique

The subject will be in supine position and the physiotherapist will position the proximal hand, taking the patient’s occipital in the palm, and the other hand under the posterior arch of the atlas, keeping the fingers extended for greater stability. With the proximal hand, the physiotherapist will position the head in a slight ventral flexion, a slight contralateral tilt and ipsilateral rotation towards the hypomobile atlanto-occipital joint, and will exert pressure with the shoulder on the patient’s forehead in a dorsal direction so that the occipital condyle slides dorsally on the atlas, which remains stable due to being supported in the distal hand (Fig. 2).

#### Pressure group procedure

The Pressure Group will receive the same protocolized treatment and the same instructions as the Control Group and will also receive 6 sessions (two sessions per week on alternate days) of the pressure maintained suboccipital inhibition technique (PMSIT) [14, 16] applied for 5 min.

**Fig. 3:**
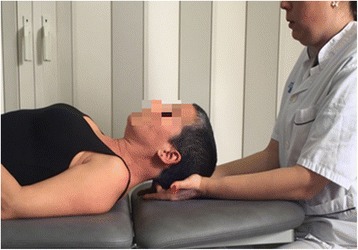
Pressure maintained suboccipital inhibition technique (PMSIT)

The subject will be in supine position and the physiotherapist will be seated at the head of the patient with the forearms resting on the table. The physiotherapist locates the suboccipital region, and applies pressure in a ventral direction in that region by flexing the metacarpophalangeal joints at 90° from fingers III-IV, while the rest of the patient head reposes on the palms of the hands, enabling the pressure to be regulated as perceived by the therapist while performing the technique (Fig. 3).

### Statistical analysis

The data analysis will be performed after the data for the entire sample has been collected, using the SPSS Stadistic® v.20.0 software package. The significance level is established at 0.05 and the limits of the confidence interval at 95%.

A descriptive analysis of the baseline characteristics of the sample will be performed. All the quantitative variables will be analysed using the Kolmogorov-Smirnov test with Lilliefors corrections, to ascertain whether they follow a normal distribution. The initial homogeneity between the groups will be also analysed (ANOVA or Kruskal-Wallis depending of the normality of data distribution). According to the results of the homogeneity and normality analysis, parametric or non-parametric statistics will be used for the within and between-group analysis.

Within-group results will be analysed using repeated measures ANOVA (when initial homogeneity and normality of data distribution were found), Friedman test (when initial homogeneity but not normality of data distribution were found), or a linear mixed model adjusted for the baseline values when not initial homogeneity were found.

Between-group results will ve analysed using ANOVA with Bonferroni post hoc statistic (when initial homogeneity and normality of data distribution were found), Kuskall-Wallis test (when initial homogeneity but not normality of data distribution were found) or a linear mixed model adjusted for the baseline values, when not inicial homogeneity were found.

The principles of intention-to-treat analysis will be used. In the event of follow-up losses, the outcome variables that have not been recorded will be completed with the last data recorded for each of these variables (Last-Observation-Carried-Forward-Analysis).

## Discussion

This study aims to compare the effectiveness of the specific manual approach to the suboccipital region, comparing between two manual techniques added to a protocolized treatment, which follows Sant Ildefons Rehabilitation Service policy, in subjects with chronic mechanical neck pain presenting restricted mobility of the upper cervical spine evaluated with the flexion-rotation test. Seventy eight subjects will be included in the study, and will be divided into three groups according to the randomization performed prior to recruitment. The variables that will be evaluated post-treatment and in the short term will be pain intensity, neck disability, cervical range of motion, headache intensity and subjective rating of clinical change.

Subjects with restricted mobility in the flexion-rotation test have been included because we want to determine whether the manual techniques considered are effective for increasing the range of motion, and which of the two techniques is more effective. Although a limitation in the range of motion is not indicative of cervical pathology, it is undoubtedly indicative of a poor mechanical function [35]. 18.7% of asymptomatic subjects have been found to possibly present restricted cervical mobility [36], and there is an association between cervicogenic headache and restricted mobility [37, 38]. As such, determining which manual technique achieves the best results for cervical mobility may be helpful in the treatment of these subjects.

There are some differences between the two manual techniques we use in this study. Although both focus in the suboccipital region, with the suboccipital inhibition technique the pressure is generated solely by the weight of the subject’s head and the physiotherapist regulates the pressure with the fingers, and it is considered a technique aimed to relax the soft tissue muscle due to the maintained pressure. Meanwhile, the translatory dorsal glide mobilization technique for the atlanto-occipital joint is considered a technique aimed to the joint, which receives cycles of dorsal pushes due to the overpressure exerted by the therapist on the subject’s forehead, in a position in which the periaticular tissue is tensioned. Unlike the suboccipital inhibition technique, the translatory dorsal glide mobilization technique causes a slight flexion of the segment. With this study we hope to clarify whether if the possible gain in mobility is due to the relaxation in the soft tissue or the gain in the sliding of the joint in segment C0-C1.

For pain intensity, the study by González et al. [16] which assesses the effectiveness of these same manual techniques on pain and blood pressure in patients with mechanical neck pain, reports that the only technique that achieves a statistically significant improvement in pain intensity is the translatory dorsal glide mobilization technique. However, this was a pilot study with a small sample size, and we hope that our study provides further evidence for the results in this variable. Based on our clinical experience, we anticipate exceeding the minimum clinically relevant difference for the VAS, which according to Carreon et al. [39] is 2.5 cm.

For the Neck Disability Index, we also expect to exceed the minimum clinically relevant difference reported in the literature, ranging from 3.5 points by Stratford et al. [40], 5 points by Pool et al. [41], 7.5 points by Carreon et al. [35] to 9.5 points by Young et al. [42].

For changes in cervical mobility, Hidalgo et al. [43] found a statistically significant increase of 17.6° in the FRT in asymptomatic subjects, with one session of the translatory dorsal glide mobilization technique. We do not know if this increase will occur in subjects with neck pain, or if the gain in mobility will persist in the short term. Furthermore, regardless of the possible gain in mobility, an aspect to consider is whether the subjects were hypomobile according to the FRT at baseline, whether they are no longer hypomobile post-treatment and if this situation persists in the short term.

Any headaches occurring and their intensity during the follow-up period will be recorded. A fall of 2.3 points in the HIT-6 has been considered clinically significant in subjects with cervicogenic headache [44], although for patients with tension headache Castien et al. [45] propose a fall of 8 points for consideration as clinically relevant.

Although changes in the patient’s medication were not encouraged in this study for ethical reasons, we anticipate consumption to be reduced, especially of medication that the subject administers on demand to control their symptoms. Only changes in the amount of medication taken by the subject in the short and medium term related to their cervical pain will be recorded.

If this study provides positive results, it will be possible to recommend that these techniques be implemented in treatment protocols for patients with neck pain, and to justify new cost-effectiveness studies, or studies of other pathological processes in the cervical region.
